# Effect of Electrocautery and Laser Treatment on the Composition and Morphology of Surface-Modified Titanium Implants

**DOI:** 10.3390/bioengineering10111251

**Published:** 2023-10-26

**Authors:** Jin-Seok Lee, Keunbada Son, Sung-Min Hwang, Young-Tak Son, Yong-Gun Kim, Jo-Young Suh, Jun Ho Hwang, Sung-Min Kwon, Jong Hoon Lee, Hyun Deok Kim, Kyu-Bok Lee, Jae-Mok Lee

**Affiliations:** 1 Department of Periodontology, School of Dentistry, Kyungpook National University, Daegu 41940, Republic of Korea; jin5586@gmail.com (J.-S.L.); lhwangl89@naver.com (S.-M.H.); periokyg@knu.ac.kr (Y.-G.K.); jysuh@knu.ac.kr (J.-Y.S.); 2 Advanced Dental Device Development Institute (A3DI), Kyungpook National University, Daegu 41940, Republic of Korea; oceanson@knu.ac.kr (K.S.); dudxkr741@naver.com (Y.-T.S.); 3 Department of Dental Science, Graduate School, Kyungpook National University, Daegu 41940, Republic of Korea; 4 Institute of Advanced Convergence Technology, Kyungpook National University, Daegu 41061, Republic of Korea; hjh@iact.or.kr (J.H.H.); sungmin@iact.or.kr (S.-M.K.); laser@knu.ac.kr (J.H.L.); 5 School of Electronics Engineering, Kyungpook National University, Daegu 41566, Republic of Korea; hdkim@knu.ac.kr; 6 Department of Prosthodontics, School of Dentistry, Kyungpook National University, Daegu 41940, Republic of Korea

**Keywords:** dental implant, electrocautery treatment, laser treatment, surface roughness

## Abstract

The purpose of this study was to investigate the effects of different peri-implantitis treatment methods (Er,Cr:YSGG laser, diode laser, and electrocautery) on various titanium implant surfaces: machined; sandblasted, large-grit, and acid-etched; and femtosecond laser-treated surfaces. Grade 4 titanium (Ti) disks, with a diameter of 10 mm and a thickness of 1 mm, were fabricated and treated using the aforementioned techniques. Subsequently, each treated group of disks underwent different peri-implantitis treatment methods: Er,Cr:YSGG laser (Biolase, Inc., Foothill Ranch, CA, USA), diode laser (Biolase, Inc., Foothill Ranch, CA, USA), and electrocautery (Ellman, Hicksville, NY, USA). Scanning electron microscopy, energy-dispersive X-ray spectroscopy, and wettability were used to characterize the chemical compositions and surfaces of the treated titanium surfaces. Significant changes in surface roughness were observed in both the electrocautery (Sa value of machined surface = 0.469, SLA surface = 1.569, femtosecond laser surface = 1.741, and *p* = 0.025) and Er,Cr:YSGG laser (Ra value of machined surface = 1.034, SLA surface = 1.380, femtosecond laser surface = 1.437, and *p* = 0.025) groups. On femtosecond laser-treated titanium implant surfaces, all three treatment methods significantly reduced the surface contact angle (control = 82.2°, diode laser = 74.3°, Er,Cr:YSGG laser = 73.8°, electrocautery = 76.2°, and *p* = 0.039). Overall, Er,Cr:YSGG laser and electrocautery treatments significantly altered the surface roughness of titanium implant surfaces. As a result of surface composition after different peri-implantitis treatment methods, relative to the diode laser and electrocautery, the Er,Cr:YSGG laser increased oxygen concentration. The most dramatic change was observed after Er:Cr;YSGG laser treatment, urging caution for clinical applications. Changes in surface composition and wettability were observed but were not statistically significant. Further research is needed to understand the biological implications of these peri-implantitis treatment methods.

## 1. Introduction

Dental implants are a robust solution for tooth replacement. However, post-implantation failures, notably due to peri-implantitis, remain a concern [1,2]. Peri-implantitis, an inflammatory disorder induced by microbial infections, leads to the degradation of the supporting alveolar bone [2]. Microorganisms engulf the surface of the implant [3], creating a bacterial biofilm that provokes an inflammatory response in adjacent soft tissues. If left untreated, progressive destruction of the marginal bone ensues [3,4]. Numerous studies highlighted the role of microbial deposition in peri-implantitis [1,2,3,4] as well as the need to eradicate bacterial biofilm for effective peri-implantitis management. However, optimal strategies to decontaminate the compromised implant surface and to rejuvenate peri-implant tissues remain elusive.

Traditional methods for removing microbial deposits from dental implants predominantly involve mechanical debridement using tools such as plastic curettes and titanium brushes [5]. Several chemical agents, including chlorhexidine and minocycline, were recommended for targeted applications [6]. Air-abrasive devices are also used to detoxify the implant surface; however, efficacy wanes for deeply situated rough implant surfaces with infrabony defects [7]. Due to their inherently bactericidal properties, lasers can be used to disinfect implant surfaces. Unlike conventional mechanical devices, lasers offer several benefits: tissue ablation, hemostasis, and decontamination [8]. Their subdued noise and vibration profiles enhance patient comfort while minimizing collateral tissue damage, reducing post-procedural discomfort, and improving prognosis [8]. Moritz et al. showed that laser interventions can drastically diminish the risk of infection and reduce the prevalence of pathogens such as *Aggregatibacter actinomycetemcomitans*, *Prevotella intermedia*, and *Porphyromonas gingivalis* [9].

Although Er:YAG and Er,Cr:YSGG lasers are primarily used for dental caries and soft tissue treatments, their hemostatic effects are limited [10]. Diode and CO_2_ lasers are suitable for soft tissue surgeries despite risks of extensive thermal repercussions [10]. The surface of peri-implantitis-afflicted implants must be cleaned without being modified. Kreisler et al. assessed how various laser types affect titanium implant surfaces and found that Nd:YAG and Ho:YAG lasers modified the surface and are therefore unsuitable for peri-implantitis treatments [11]. Likewise, Romanos et al., observed deleterious effects of Nd:YAG lasers on implant surfaces [12]. Conversely, in vitro studies showed the antimicrobial efficacy of Er:YAG, CO_2_, and diode lasers against bacteria on titanium surfaces [13]. Notably, CO_2_ and diode lasers were found to be benign against titanium, but Er:YAG lasers eliminated surface microorganisms without modifying the surface [14,15].

The architecture of the implant surface significantly influences osseointegration [16]. Therefore, a variety of surface treatments were proposed to optimize implant success rates and osseointegration [17,18], including standalone sandblasting and hybrid techniques incorporating acid etching. Notably, the sandblasted, large-grit, and acid-etched (SLA) surface treatment enhances cellular proliferation and bone implant juxtapositions while possibly reducing treatment timelines [19,20]. Buser et al., showed how the SLA surface fosters osseointegration in porcine models [21]; their subsequent studies echoed these findings and the efficacy of the SLA surface in torque retention [22]. Vorobyev et al. illustrated the potential of the femtosecond laser to fabricate intricate nanostructures on titanium surfaces that support cell adhesion and proliferation [23,24].

A previous study sought to identify optimal power and exposure durations for diode lasers to cleanse titanium disc surfaces sans damage [25]. This study evaluated surface alterations on a variety of implant surfaces post-laser periodontal treatments, particularly Er,Cr:YSGG and diode lasers. Electrocautery, another viable option for peri-implant tissue management, was also assessed. Within the vast landscape of implant surfaces, we compared the less characterized femtosecond laser-treated surfaces with machined surfaces and SLA surface implants. The purpose of this study was to investigate the effects of different peri-implantitis treatment methods (Er,Cr:YSGG laser, diode laser, and electrocautery) on various titanium implant surfaces: machined; sandblasted, large-grit, and acid-etched; and femtosecond laser-treated surfaces. The null hypothesis of this study was that different peri-implantitis treatment methods (Er, Cr:YSGG laser, diode laser, and electrocautery) do not effect various titanium implant surfaces (machined; sandblasted, large-grit, and acid-etched; and femtosecond laser-treated surfaces).

## 2. Materials and Methods

### 2.1. Implant Fixture Preparation and Surface Treatment of Titanium Disk

- Machined surface group: Grade 4 titanium discs with a diameter of 10 mm and a thickness of 1 mm were fabricated by a dental implant manufacturer (DENTIS, Daegu, Republic of Korea) and processed using a CNC milling machine. The discs were composed of 0.08% carbon (C), 0.5% iron (Fe), 0.015% hydrogen (H), 0.05% nitrogen (N), 0.40% oxygen (O), and 98.9% titanium (Ti).
- SLA surface group: Titanium discs were sandblasted with a large volume of alumina particles (250–500 μm) at a pressure of 4 bar for 20 min. They were then oxidized using a mixture of HCl (65%) and H_2_SO_4_ (9%) (in a 1:1 volume ratio) at 60 °C for 30 min, followed by rinsing with distilled water and air-drying at ambient conditions.
- Femtosecond laser surface group: Titanium disc surfaces were treated using a femtosecond laser operating at a wavelength of 343 nm, a scanning speed of 10 mm/s, and a repetition frequency of 200 kHz. As a result, lines were patterned at intervals of 50 μm on the titanium (Table 1).

**Figure 1:**
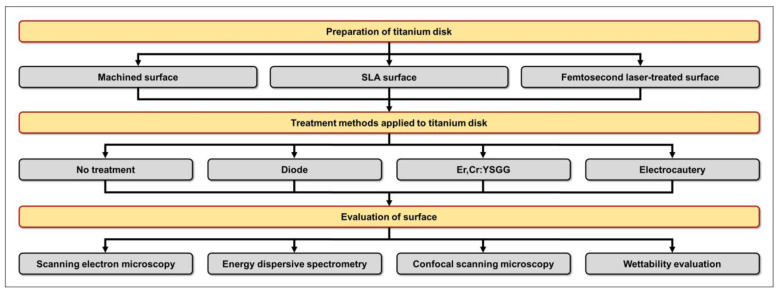
Schematic depicting the chronology of titanium disk preparation, treatment, and evaluation.

After conducting pilot experiments prior to the current study, we determined that 3 samples per group would be appropriate based on the following results obtained using power analysis software (G*Power v3.1.9.2; Heinrich Heine University, Dusseldorf, Germany): actual power = 81.7%; and power = 80%. To minimize potential investigator-induced variations, experienced researchers performed repeated experiments under identical conditions, and all utilized devices were calibrated in accordance with the manufacturer’s instructions prior to use. Additionally, all experiments were conducted under consistent conditions (Figure 1).

### 2.2. Dental Laser and Electrocautery

All peri-implantitis treatment methods in the present study were performed by one investigator (J.-S.L.) skilled in the technique. Three dental diode lasers ranging from 940 nm to 980 nm were selected based on a previous study [24]. Here, two types of lasers and an electrocautery device were used:

- Epic 10^TM^ (Biolase, Inc., Foothill Ranch, CA, USA) for diode laser at a wavelength of 940 nm;
- Waterlase iPlus^®^ (Biolase, Inc., Foothill Ranch, CA, USA) for the Er,Cr:YSGG laser at a wavelength of 2780 nm;
- Bovie Surgitron FfpF (Ellman, Hicksville, NY, USA) for electrocautery. Ten-second treatments were administered as described in Table 2.

**Figure 2:**
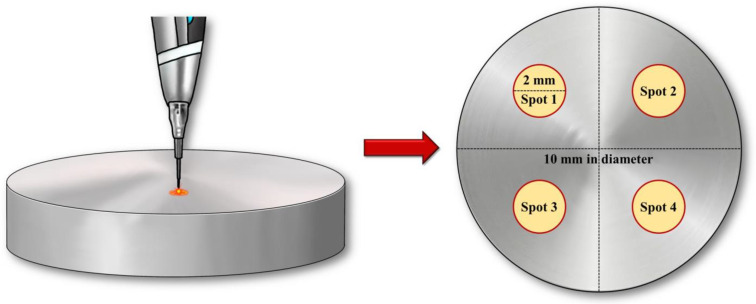
Schematic of laser irradiation points on titanium disks. Spot 1: control. Spot 2: Diode laser. Spot 3: Er,Cr:YSGG Laser. Spot 4: Electrocautery.

The laser and electrocautery tips were chosen to be identical in size; they remained static and made direct perpendicular contact with the titanium disc surface for 10 s (Figure 2). All lasers operated in continuous wave mode using a flexible fiber, generating a focused spot whose diameter was 0.4 mm. The laser parameters are listed in Table 2.

### 2.3. Surface Roughness Assessment via Scanning Electron and Confocal Scanning Microscopy

Titanium discs were thoroughly cleaned in an ultrasonic bath using a mixture of acetone, ethanol, and distilled water (1:1:1 *w*/*v*/*v*) for 15 min at each step. The discs were then oven-dried at 60 °C for 2 h and analyzed using a scanning electron microscope (SEM; Hitachi SU8230; Hitachi, Tokyo, Japan) at an acceleration voltage of 5 kV and 100× magnification.

Confocal microscopy images were captured at 10× magnification using a laser scanning confocal microscope (LEXT OLS4100; Olympus, Tokyo, Japan). The surface roughness of the treated titanium discs was quantified using the arithmetic mean roughness (Ra) and arithmetical mean height (Sa) derived from the microscopy images. Each sample was measured thrice to evaluate how each surface treatment affected implant texture.

### 2.4. Wettability Assessment

Titanium disc wettability was measured using a contact angle goniometer (Phoenix-MT; SEO, Suwon, Republic of Korea) and the following procedure was conducted as prior study [26]. A two-microliter droplet of deionized water was placed on each sample using a micro syringe. The contact angle between the liquid droplet and the solid disc surface was measured within 10 s. This procedure was repeated five times for every sample to obtain an average contact angle.

### 2.5. Energy-Dispersive Spectrometry

Energy-dispersive X-ray spectroscopy (EDS) was conducted using a field emission SEM coupled with an Oxford ULTIM MAX 100 (SU8230, Hitachi, Ltd., Tokyo, Japan) to characterize the chemical properties of the scanned disks. This method provided atomic and weight percentage ratios for titanium, oxygen, and carbon.

### 2.6. Statistical Analysis

Non-parametric statistical evaluations were conducted using SPSS software (version 26; IBM Corp., Armonk, NY, USA). The Kruskal–Wallis H test was performed (α = 0.05) to assess differences in surface roughness and contact angles across the three implant surface treatments. In the case of significant differences, subsequent pairwise comparisons were executed using post-hoc tests and the Bonferroni correction. Significant differences between the implant surface treatments were denoted using uppercase letters (α = 0.05).

The Kruskal–Wallis H test was also used (α = 0.05) to assess differences in wettability across the implant surface treatments. As stated above, pairwise examinations were conducted with the Bonferroni correction. Significant differences between the implant surface treatments were denoted using uppercase letters (α = 0.05).

## 3. Results

### 3.1. Surface Roughness Measured by Scanning Electron Microscopy (SEM)

**Figure 3:**
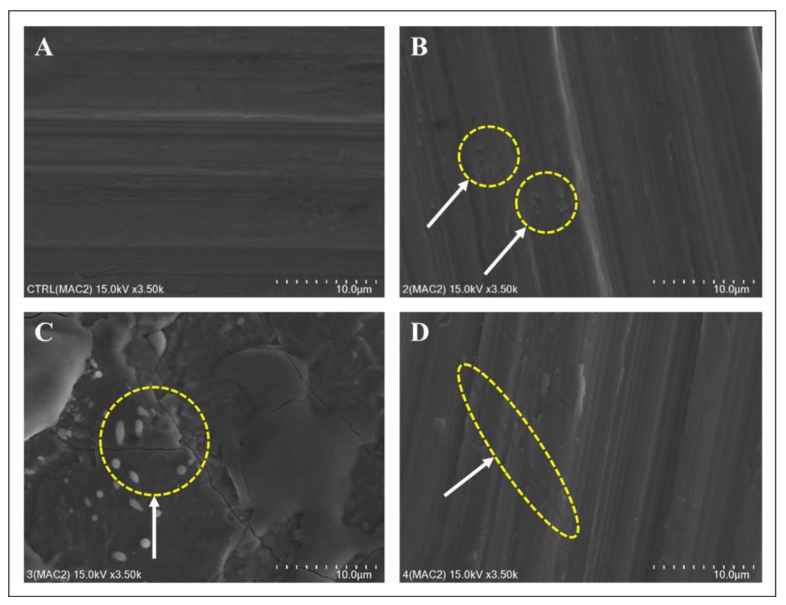
Surface changes following laser and heat treatment of machined surfaces. (**A**) Control: smooth surface. (**B**) Diode laser: pits observed after diode laser treatment. (**C**) Er,Cr:YSGG treatment: Cracks were observed along with an increase in the surface roughness of the titanium. Some molten surfaces were observed. (**D**) Electrocautery: minor surface changes were observed after electrocautery treatment.

**Figure 4:**
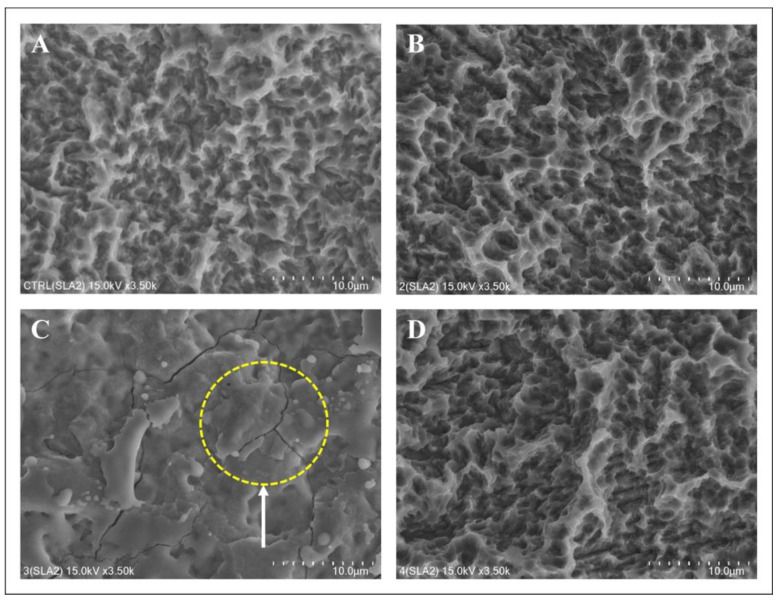
Surface changes following laser and heat treatment of the SLA surface. (**A**) Control: SEM image of the SLA surface. Large-grit sand blasting and acid etching generated macro-roughness and micro-roughness, respectively. (**B**) Diode laser: no changes were observed after treatment. (**C**) Er,Cr:YSGG laser: typical cracks and a molten area were observed on the titanium surface. (**D**) Electrocautery: no changes were observed after treatment.

**Figure 5:**
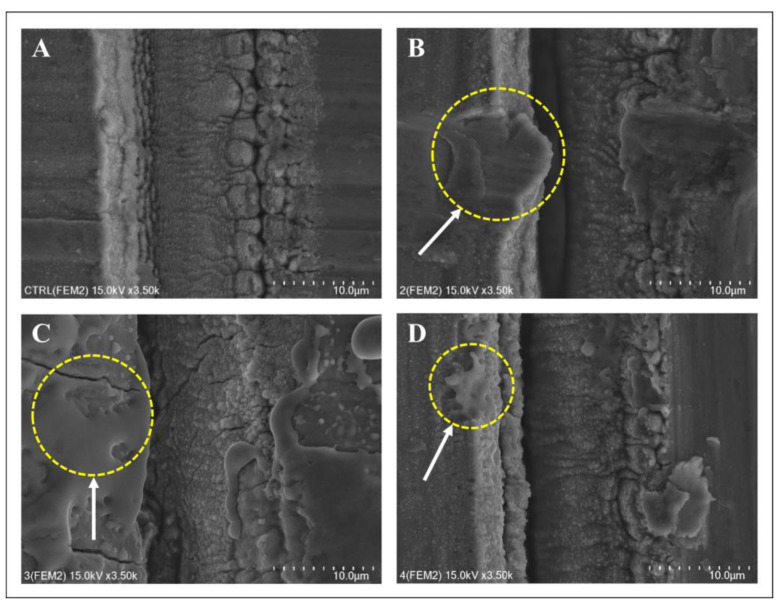
Surface changes following laser and heat treatment on the femtosecond laser-treated surface. (**A**) Control: SEM image of the femtosecond laser-treated surface. After surface treatment, the roughness of the titanium surface increased and formed a lattice pattern. (**B**) Diode laser: after treatment, some molten areas and minor surface deformation were observed. (**C**) Er,Cr:YSGG laser: Molten area with numerous cracks was observed on the titanium surface. (**D**) Electrocautery: A slight molten area was observed after treatment with a Bovie Surgitron.

SEM images were acquired after treating the surface of each disc (Figure 3, Figure 4 and Figure 5). The structure of the machined surface disc was consistent (Figure 3A), unlike the SLA group, whose rougher surface featured microgrooves from sandblasting and acid etching (Figure 4A). The femtosecond laser-treated surface exhibited recurring linear microgrooves (Figure 5A). Most discs showed no substantial alterations after combined treatment with the laser and heat, yet the Er,Cr:YSGG laser overall significantly changed the surface roughness. The Er,Cr:YSGG laser formed a rugged texture with distinct spikes and deep pits on the SLA surface (Figure 4C). Conversely, patterns indicative of melting were found near the irradiated zone on the machined surface, mirroring those on the femtosecond laser-treated surface (Figure 3C and Figure 5C).

### 3.2. Composition Post-Laser and Electrocautery Application

Table 3, Table 4 and Table 5 list the shifts in chemical composition detected via EDS analysis for each disc. The compositional differences between the untreated machined and SLA surfaces were negligible. In contrast, the femtosecond laser-treated surface had a higher oxygen fraction but lower titanium content. Relative to the diode laser and electrocautery, the Er,Cr:YSGG laser increased oxygen concentration and reduced titanium content.

### 3.3. Surface Roughness Assessed by Confocal Microscopy

**Figure 6:**
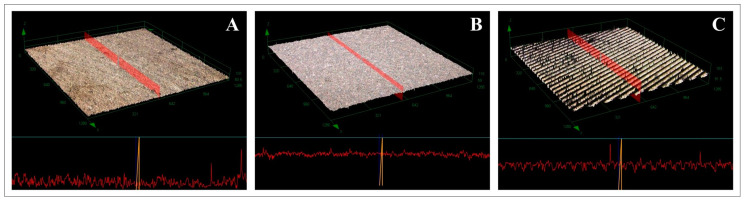
Confocal laser scanning microscopy images depicting the surface morphologies of the (**A**) machined group, (**B**) SLA group, and (**C**) femtosecond laser-treated group.

Surface profiles and attributes across groups are shown in Figure 6, and Table 6 and Table 7 present qualitative roughness parameters. Notably, both SLA and femtosecond laser treatments yielded superior Ra and Sa values relative to the machined surface. After treatment with the Er,Cr:YSGG laser, the roughness of the SLA and femtosecond laser-treated disks was greater than that of the machined surface (*p* < 0.05). Furthermore, according to Sa values, the electrocautery-treated surfaces manifested were rougher than the others (*p* < 0.05). No other significant differences were detected in surface roughness (*p* > 0.05).

### 3.4. Wettability Test

**Figure 7:**
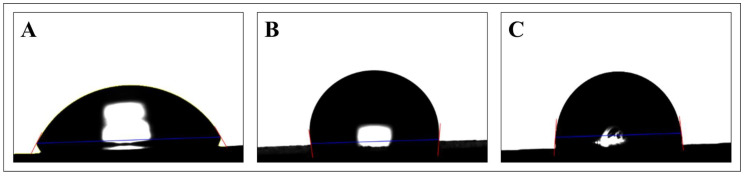
Representative surface morphology image and wettability test. (**A**) Machined group, (**B**) SLA group, and (**C**) femtosecond laser-treated group.

Surface treatments modified the contact angles (Figure 7 and Table 8) and therefore the wettability of the titanium surface. Both SLA and femtosecond laser treatments increased the hydrophilicity of the surface; the former yielded a slightly greater contact angle. Laser treatment and electrocautery reduced the contact angle with disparities observed primarily in the femtosecond laser group. Overall, the contact angle was modulated the most and the least by the Er,Cr:YSGG laser and electrocautery, respectively. 

## 4. Discussion

Implant-related infections such as peri-implantitis and mucositis often lead to implant failure [3,4]. Therefore, this study initially aimed to investigate the effects of laser and electrocautery, which are currently used for peri-implantitis treatment, on the titanium surface. Additionally, we sought to determine if there were any differences in treatment outcomes based on the type of implant surface. Despite the relatively minimal impact of peri-implantitis treatment using lasers on titanium implant surfaces, we anticipated variations based on the type of laser used in the experiment. We also expected different responses when applying lasers to three different implant surfaces, and to investigate this, we analyzed surface roughness, chemical composition, and wettability. Ultimately, while there were differences observed in roughness analysis using Ra and Sa depending on the type of implant surface, these differences did not show statistically significant results. However, there were cases where statistically significant differences were observed depending on the type of laser used, specifically with the Er,Cr:YSGG laser (Ra) and electrocautery (Sa). In the wettability test, the use of the Er,Cr:YSGG laser on femtosecond laser-treated surfaces showed statistically significant differences compared to other treatment methods.

The implant surfaces were characterized qualitatively and quantitatively using SEM and confocal microscopy. Treatment with the Er,Cr:YSGG laser at a modest power of 2 W for 10 s significantly altered the implant surface, which was also shown previously by Park et al., especially beyond the 3 W power threshold [27]. These results urge caution when using Er,Cr:YSGG lasers in clinical settings. Conversely, the diode laser only slightly altered the surface, corroborating findings from Lollobrigida et al. [28,29] and others [25]. A 10 s treatment in continuous wave mode negligibly altered the surface of a small disk, consistent with Castro [28,29]. Although the diode laser only slightly altered the surface, its decontamination efficiency may differ and should be explored in a future study. Additionally, the skill of the operator administering the laser and the variations in laser parameters can introduce differences in the results. It is also crucial to consider the heat generation resulting from the use of laser equipment. While this heat generation may aid in contamination removal, a significant temperature rise can potentially affect the surrounding teeth and bone formation. Further studies are needed to account for and explore these effects. 

EDS was used to characterize the chemical compositions of the disks after surface treatments. Specifically, the machined and SLA surfaces displayed elevated titanium (Ti) and reduced oxygen (O) levels, whereas the femtosecond laser-treated surface showed enhanced oxygen levels. Given titanium’s tendency to bond with atmospheric elements such as carbon (C), oxygen (O), and nitrogen (N), an oxidized titanium surface results in a titanium oxide (TiO_2_) layer. Augmented polarization enhances oxygen levels, promoting the formation of the TiO_2_ layer [30,31,32]. A pronounced TiO_2_ layer on the femtosecond laser-treated surface suggested enhanced biocompatibility that could enhance cellular responses to the implant surface. When treated with the Er,Cr:YSGG laser, there was a common trend of increased oxygen levels, and it is believed that this result also occurred due to surface oxidation reactions. In the research of Scarano et al., the irradiation of the SLA disk surface with Er:YAG laser depicted similar results, leading to an increase in TiO_2_ layer [33]. Additionally, the experiment of Ercan et al. showed treatment with the Er,Cr:YSGG laser at settings above 2 W resulted in an increase in oxygen levels and a decrease in titanium levels due to oxidation [34,35]. The effects of the chemical composition of the disks on cell and tissue behaviors warrant comprehensive in vitro and in vivo studies.

Contact angles were measured to determine how various treatments affect surface wettability [36,37,38,39,40,41,42,43]. After implantation, interactions between implants and fluids affect the tissue microenvironment as well as the surface of the implant. MacDonald’s research showed that implants with higher wettability promote osseointegration [44]. Our data suggest that femtosecond laser-treated surfaces may be more favorable than SLA surfaces for osseointegration by increasing hydrophilicity.

After exploring how laser treatment and electrocautery affect peri-implantitis treatment, Ra and Sa values revealed minimal differences between groups. Therefore, the resistance of specific implant surfaces to laser-induced changes remains unclear. However, the potential benefits of femtosecond laser-treated surfaces, such as enhanced osseointegration due to complex surface compositions [36] and the flexible modification of surface patterns across scales, warrant further consideration.

Ultimately, the degree of contamination on the titanium surface determines mechanical stability and osteoinductive properties [40]. Even if chemically treated implant surfaces remained uncontaminated, mechanical processes often introduce foreign substances [41]. Gaggl et al., found that femtosecond laser applications optimize surface structure without introducing contaminants, which agrees with our findings [42]. Higher surface purity may enhance osseointegration and therefore implant stability. Cunha et al., observed reduced *S. aureus* adhesion and biofilm formation on femtosecond laser-treated implants [43]. Thus, femtosecond laser texturing could render titanium implants antimicrobial, mitigating the risks associated with implant infections. While femtosecond laser texturing may reduce biofilm formation associated with peri-implantitis, the present study focused solely on evaluating the morphology of implant surfaces that underwent femtosecond laser texturing and were then subjected to different peri-implantitis treatment methods. Future research should apply these peri-implantitis treatment methods and assess the attachment levels of various biofilm types to determine the impact of post-treatment modified implant surfaces on biofilm formation.

This study has several limitations. First, unlike mechanical treatment, the area of application on the surface is very small, resulting in a relatively minimal impact. Additionally, there is a limitation in that the output and application times of the laser used in the study are not diverse, but restricted to one setting. These issues could potentially be addressed by creating larger specimens for experimentation, applying the laser to multiple areas, and refining the laser settings. Furthermore, due to the nature of in vitro studies, the evaluation of the materials used in the experiment itself constitutes a significant portion of the research. Although our study characterized the effects of various implant decontamination strategies on surface properties, clinical validations are needed. Future studies should explore the resistance of surface modifications to detoxification, particularly the implications of laser-induced changes for toxin elimination and biocompatibility. In previous research, the adhesion of osteoblasts to modified implant surfaces was investigated to assess osseointegration after specific treatments [18]. Moreover, other studies evaluated the attachment of biofilms to altered surfaces to determine the influence of modified implant surfaces on peri-implantitis [2,14]. The present study qualitatively and quantitatively analyzed the surface roughness and morphology following different peri-implantitis treatment methods, including Er,Cr:YSGG laser, diode laser, and electrocautery. Future research is needed to evaluate the adhesion of osteoblasts and biofilms, further elucidating the effects of modified implant surfaces on osseointegration and peri-implantitis.

## 5. Conclusions

This research used SEM and EDS to determine how laser treatment and electrocautery affect the surfaces of machined, SLA, and femtosecond laser-treated titanium. All treatment modalities influenced roughness parameters on each of the tested surfaces, but exposure to the Er,Cr;YSGG laser induced the most significant changes, and therefore, warrants caution for its use in clinical settings. Though not statistically significant, post-intervention changes in composition and wettability were also observed. Notably, the surfaces of femtosecond laser-treated surfaces paralleled those of traditional titanium surfaces. Additional studies are needed to better understand the biological effects of decontamination and its impact on cellular dynamics.

## Figures and Tables

**Table 1:** Parameters for femtosecond laser surface treatment.

Laser Type	Wavelength (nm)	Pulse Duration	Power (W)	Repetition (kHz)	Speed (mm/sec)	Lens
Femtosecond laser	343	<400 fs	2~3	200	10	×10

**Table 2:** Treatment modalities, energy, frequency, and duration applied in this study.

Spot	Treatment Type	Power (W)	Energy (mJ)	Frequency (Hz)	Total Time (sec)
1	Control	-	-	-	-
2	Diode laser	2.0	100	10	10
3	Er,Cr:YSGG Laser	2.0	300	10	10
4	Electrocautery	30	-	10	10

**Table 3:** Composition of the machined surface disk after laser treatment and electrocautery.

Surface Type	Composition		
	C	O	Ti
Control	4.37	15.15	80.48
Diode	4.91	14.84	80.25
Er,Cr:YSGG	2.36	62.44	35.20
Electrocautery	4.54	18.59	76.87

**Table 4:** Composition of the SLA surface disk after laser treatment and electrocautery.

Surface Type	Composition		
	C	O	Ti
Control	4.54	14.58	80.89
Diode	2.60	16.20	81.20
Er,Cr:YSGG	1.63	63.36	35.01
Electrocautery	2.93	16.75	80.32

**Table 5:** Composition of the SLA surface disk after laser treatment and electrocautery.

Surface Type	Composition		
	C	O	Ti
Control	5.79	46.19	48.01
Diode	17.19	45.66	37.15
Er,Cr:YSGG	6.06	57.48	36.47
Electrocautery	12.65	44.75	42.60

**Table 6:** Roughness (Ra value) of each implant after surface treatment.

Treatment Type	Surface Type	Mean	95% Confidence Interval		*p*	Comparison
			Lower	Upper		
Control	Machined	0.580	0.050	0.523	0.0714	
	SLA	1.316	0.353	0.916		
	Femto	1.534	0.300	1.194		
Diode	Machined	0.657	0.132	0.508	0.0714	
	SLA	1.247	0.208	1.010		
	Femto	1.342	0.356	0.938		
Er,Cr:YSGG	Machined	1.034	0.057	0.970	0.025 *	A
	SLA	1.380	0.273	1.071		B
	Femto	1.473	0.334	1.095		C
Electrocautery	Machined	0.633	0.0901	0.531	0.05	
	SLA	1.632	0.545	1.014		
	Femto	1.714	0.338	1.332		

Statistically relevant *p* < 0.05 is marked with *. A, B, and C indicate significant differences via pairwise comparison using Bonferroni correction (*p* < 0.05).

**Table 7:** Roughness (Sa value) of each implant after surface treatment.

Treatment Type	Surface Type	Mean	95% Confidence Interval		*p*	Comparison
			Lower	Upper		
Control	Machined	0.192	0.231	−0.069	0.0714	
	SLA	1.485	0.199	1.260		
	Femto	1.551	0.304	1.206		
Diode	Machined	0.251	0.319	−0.110	0.1	
	SLA	1.196	0.402	0.741		
	Femto	1.433	0.374	1.009		
Er,Cr:YSGG	Machined	0.771	0.452	0.258	0.0714	
	SLA	1.552	0.244	1.275		
	Femto	1.735	0.538	1.125		
Electrocautery	Machined	0.469	0.316	0.111	0.025 *	A
	SLA	1.569	0.276	1.256		B
	Femto	1.741	0.321	1.377		C

Statistically relevant *p* < 0.05 is marked with *. A, B, and C indicate significant differences via pairwise comparison using Bonferroni correction (*p* < 0.05).

**Table 8:** Surface contact angle of each treated surface.

Surface Type	Treatment Type	Mean	95% Confidence Interval		*p*	Comparison
			Lower	Upper		
Machined	Control	69.9	6.4	66.3	0.124	
	Diode	71.9	4.5	69.4		
	Er,Cr:YSGG	68.2	5.6	65.1		
	Bovie	67.5	7.0	63.6		
SLA	Control	86.8	21.7	74.8	0.098	
	Diode	83.0	14.2	75.1		
	Er,Cr:YSGG	84.1	16.0	75.2		
	Bovie	82.9	16.2	74.0		
Femto	Control	82.2	8.1	77.6	0.039 *	A
	Diode	74.3	12.	67.2		A
	Er,Cr:YSGG	73.8	6.3	70.3		B
	Bovie	76.2	12.2	69.4		A

Statistically relevant *p* < 0.05 is marked with *. A and B indicate significant differences via pairwise comparison using Bonferroni correction (*p* < 0.05).

## Data Availability

The datasets used and/or analyzed during the current study are available from the corresponding author upon reasonable request.
